# Protocol for an AlCl_3_-induced Alzheimer’s model in zebrafish larvae with optimized pH and behavioral assessment

**DOI:** 10.1016/j.xpro.2026.104657

**Published:** 2026-06-26

**Authors:** Zequan Lin, Lufang Zou, Wei Li, Lijia Pan, Jinchen Zhang, Ruiqin Hu, Liangbiao Chen

**Affiliations:** 1 Key Laboratory of Aquacultural Resources and Utilization, Ministry of Education, College of Fisheries and Life Science, Shanghai Ocean University, Shanghai 201306, China; 2 International Research Center for Marine Biosciences, Ministry of Science and Technology, Shanghai Ocean University, Shanghai 201306, China

**Keywords:** Behavior, Cognitive Neuroscience, Model Organisms

## Abstract

Alzheimer’s disease (AD) is a progressive neurodegenerative disorder that impairs cognition and memory. Current protocols for establishing AD models in zebrafish (*Danio rerio*) larvae are limited and lack comprehensive detail. We present a refined zebrafish model, optimizing pH control of AlCl_3_ solutions and improving the behavioral assays to ensure a consistent AD-like phenotype. We describe steps for breeding embryos, testing behavior, selecting and treating larvae, and analyzing data. This protocol facilitates high-throughput screening of potential therapeutic drugs and genes for AD.

## Highlights

• Steps for preparing pH-controlled AlCl_3_ solution to induce Alzheimer’s-like pathology
• Instructions for selecting and treating zebrafish larvae with AlCl_3_ from 72 to 144 hpf
• Guidance on conducting and analyzing light/dark locomotion behavioral assays

## Abstract (editor highlights)

Publisher’s note: Undertaking any experimental protocol requires adherence to local institutional guidelines for laboratory safety and ethics.

## Graphical abstract

**Figure:**
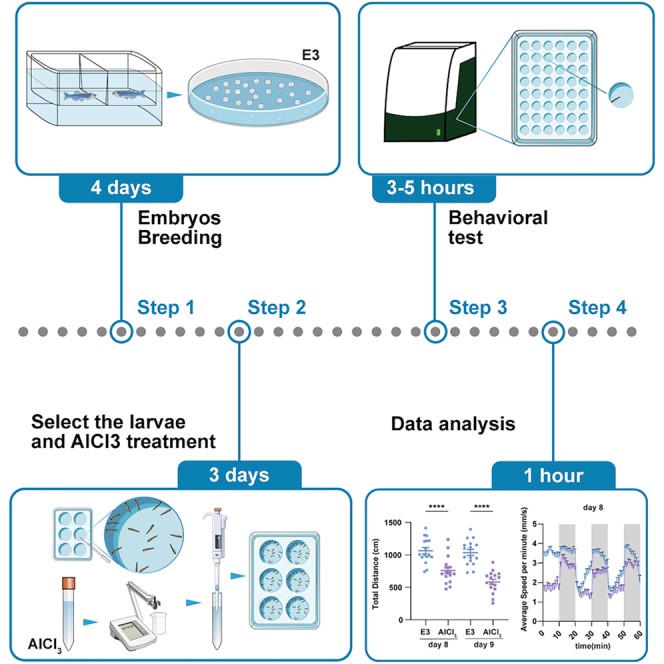

## Before you begin

To screen for gene or small molecule drugs that may treat Alzheimer’s disease, various animal models, such as nematodes, fruit flies, zebrafish, mice and rats are used.^1^ Nematodes and fruit flies are cost-effective and have short life cycles but lack complex neurological features.^2^^,^^3^ Mice and rats, though commonly used, are expensive and often require several months to over a year to develop Alzheimer’s disease-related pathology.^4^ In contrast, zebrafish (*Danio rerio*) larvae are increasingly popular due to their short breeding cycle, low cost, and neurological similarity to humans. Their transparent embryos also facilitate microinjection and fluorescence-based assays. Despite the widespread use of AlCl_3_ in modeling, the detailed protocols, including exposure duration, AlCl_3_ concentration, and behavioral testing timelines, vary considerably among studies, which hinders cross-study comparison and reproducibility.^1^^,^^5^^,^^6^^,^^7^^,^^8^^,^^9^ Previous studies have shown that in zebrafish larval exposure models, 100–200 μM AlCl_3_ can induce Alzheimer’s disease-related phenotypes, such as cognitive impairment and neuronal apoptosis.^5^^,^^6^^,^^7^^,^^10^^,^^11^ Among these, 150 μM is a concentration commonly used in multiple studies. However, aluminum ions induce AD-like pathology, but AlCl_3_ hydrolysis lowers pH of the solution, which can harm zebrafish larvae.^12^^,^^13^ In our preliminary experiments, the modeling effect of 100 μM AlCl_3_ was unstable, while 150 μM AlCl_3_ caused significant mortality and malformations in zebrafish larvae. Therefore, we selected 150 μM AlCl_3_ as the optimal concentration for AD modeling, but it is essential to carefully regulate pH to ensure it is suitable for larval growth and development prior to administering AlCl_3_ treatments. This protocol provides detailed steps and solution parameters to ensure reproducibility and consistency in AD modeling.

### Innovation

This protocol provides a standardized workflow for establishing an Alzheimer’s disease (AD) model in zebrafish larvae using aluminum chloride (AlCl_3_), addressing the limitations of existing methods in reproducibility and experimental stability. The key innovation lies in the precise control of the AlCl_3_ solution pH (4.8–4.9), which balances aluminum ion bioavailability with larval survival. Unlike conventional methods that overlook hydrolysis-induced acidification, this protocol maintains stable treatment conditions through controlled NaOH titration and daily preparation of fresh solutions, thereby reducing mortality and minimizing non-specific stress effects. Furthermore, by introducing a standardized light/dark cycling paradigm and a defined circadian testing window, this protocol improves the sensitivity for detecting locomotor and rhythm-related phenotypes. Overall, this protocol establishes a robust, reproducible, and scalable zebrafish-based AD modeling platform suitable for high-throughput AD drug screening.

### Institutional permissions

For the procedure described here, the AB zebrafish line was used. All handling of fish was conducted in accordance with the guidelines for the care and use of animals for scientific purposes, as set by the Institutional Animal Care and Use Committee (IACUC) of Shanghai Ocean University (SHOU), Shanghai, China. This research was approved by IACUC (SHOU-DW-2021-061) of SHOU.

### Preparation of stock solutions

**Timing: 1 h**

1. Prepare the 10× E3 medium, 1M AlCl_3_ solution and 1M NaOH solution (recipe to be found in materials and equipment).

**CRITICAL:** Instead of anhydrous aluminum chloride(AlCl_3_), aluminum chloride hexahydrate (AlCl_3_·6H_2_O) is preferred. AlCl_3_ is hygroscopic and absorbs moisture from the air during weighing, leading to errors in measuring its mass. Additionally, when dissolved in water, AlCl_3_ releases a large amount of heat and white fumes.

### Zebrafish breeding

**Timing: 1–2 h per day for 4 consecutive days (days 1–4)**

2. Set up a breeding tank with male and female fish, separated by a barrier.
3. Remove the barriers for all tanks at 9:00 A.M. the following morning.
4. Collect the fertilized embryos and place a maximum of 150 embryos in a Petri dish containing 1× E3 medium. Incubate the embryos at 28°C under a diurnal light cycle of 14 hours of light and 10 h of darkness, with lights on at 8:00 A.M. and off at 10:00 P.M.
5. On the first three days, carefully remove any malformed, dead, or unfertilized embryos, and replace with fresh 1× E3 medium.

***Note:*** Zebrafish are maintained in a recirculating aquaculture system using 3 L tanks, with no more than 20 adult fish per tank. Males and females are housed separately. The water temperature is maintained at 28 ± 1 °C, with a pH of 7.0–7.5 and conductivity between 300–800 μS. Water quality parameters are regularly monitored and kept stable. The light cycle is set to 14 h light/10 h dark, with lights on at 8:00 A.M. and off at 10:00 P.M. Adult fish receive feeding twice daily at 9:00 A.M. and 5:00 P.M. with brine shrimp nauplii. Larvae receive feeding at the same times with paramecia.

## Key resources table

**Table:** 

REAGENT or RESOURCE	SOURCE	IDENTIFIER
**Chemicals, peptides, and recombinant proteins**		

NaCl (sodium chloride)	Sangon Biotech	Cat# A610476
KCl (potassium chloride)	Sangon Biotech	Cat# A610440
MgSO_4_·7H_2_O (magnesium sulfate heptahydrate)	Sangon Biotech	Cat# A610329
CaCl_2_·2H_2_O (calcium dichloride)	Sangon Biotech	Cat# A501331
AlCl_3_·6H_2_O (crystalline aluminum chloride)	Aladdin	Cat# A112511

**Experimental models: Organisms/strains**		

Zebrafish (*Danio rerio*): wild type AB strain larvae; sex not specified yet	Shanghai Ocean University	N/A

**Software and algorithms**		

Prism 9	GraphPad	https://www.GraphPad.com
EthoVision XT (version 15.0.1418)	Noldus	https://noldus.com/daniovision
ZEN	Zeiss	www.zeiss.com

**Other**		

6-well Clear TC-treated Multiple Well Plates	Corning	Cat# 3516
48-well Clear TC-treated Multiple Well Plates	Corning	Cat# 3548
96-well Receiver Plate	Corning	Cat# 3382
P1000 Mechanical Pipette	Eppendorf	Cat# 3123000063
P200 Mechanical Pipette	Eppendorf	Cat# 3123000055
P20 Mechanical Pipette	Eppendorf	Cat# 3123000039
P1000 Pipette tips	GeneBrick	Cat# GB010010
P200 Pipette tips	GeneBrick	Cat# GB010200
P20 Pipette tips	GeneBrick	Cat# GB011000
15mL Centrifuge Tube	Labselect	Cat# CT-002-15A
1.5mL Centrifuge Tube	Sangon Biotech	Cat# F600620
Versatile stereo microscope	Nikon	Cat# SMZ800
DanioVision	Noldus	https://noldus.com/daniovision
Thermostatic incubator	Panasonic	Cat# MIR-254-PC
pH meter	Mettler Toledo	Cat# S 20

## Materials and equipment

**Table:** 1M NaOH stock solution

Reagent	Final concentration	Amount
NaOH	1 M	2.0 g
Milli-Q water	N/A	Up to 50 mL
**Total**	**N/A**	**50 mL**

0.1M NaOH solution - 100 μL 1 M NaOH stock solution in 900 μL Milli-Q water.

***Note:*** Store at 4 °C for up to 3 months.

**Table:** 10× E3 medium stock solution

Reagent	Final concentration	Amount
NaCl	5 mM	2.9 g
KCl	0.17 mM	0.13 g
MgSO_4_·7H_2_O	0.33 mM	0.81 g
CaCl_2_·2H_2_O	0.33 mM	0.49 g
Milli-Q water	N/A	Up to 1L
**Total**	**N/A**	**1L**

Adjust pH to 7.2 with 0.1 M NaOH solution and sterilize it by autoclaving.

***Note:*** Store at room temperature (22 °C) for up to 3 months.

1× E3 medium - 50 mL 10× E3 medium in 450 mL Milli-Q water.

***Note:*** Compared to recirculating aquaculture system (RAS) culture water, E3 medium is a better choice. E3 medium provides a stable water quality and ionic environment, reducing the risk of microbial contamination.

**Table:** 1 M AlCl_3_ stock solution

Reagent	Final concentration	Amount
AlCl_3_·6H_2_O	1 M	2.4143 g
Milli-Q water	N/A	8.9200 mL
**Total**	**N/A**	**10 mL**

***Note:*** Store at 4 °C for up to 3 months.

0.1 M AlCl_3_ solution - 20 μL 1 M AlCl_3_ stock solution in 180 μL Milli-Q water.

150 μM AlCl_3_ solution - 15 μL 0.1 M AlCl_3_ solution in 10 mL 1× E3 medium working solution.

Adjust the pH of 150 μM AlCl_3_ to 4.8–4.9 with 0.1 M NaOH solution.

**Figure 1:**
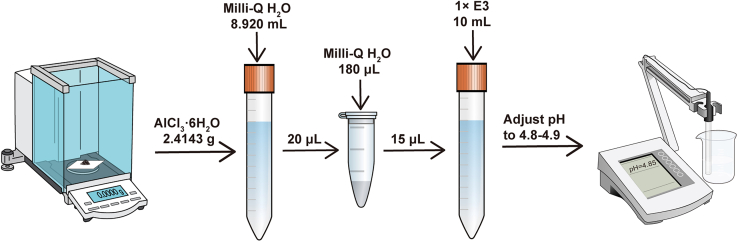
Preparation of AlCl_3_ solution used for modeling

Figure 1 is the procedure for preparing the working solution for Alzheimer’s disease modeling.

***Note:*** The prepared 0.1 M AlCl_3_ solution can be used for up to 5 days. The 150 μM AlCl_3_ solution (pH 4.8–4.9) should be prepared fresh daily, as the reaction of atmospheric carbon dioxide with water can lower the pH of the solution over time.

***Optional:*** To assess a potential therapeutic drug for Alzheimer's disease, the drug should be added after pH adjustment to ensure precise experimental conditions. However, it is essential to ensure that the addition of the drug does not significantly alter the pH. The drug concentration should be adjusted based on the specific experimental requirements while ensuring the concentration is within a safe range for zebrafish larvae, as determined by preliminary toxicity tests or literature references.

**Figure 2:**
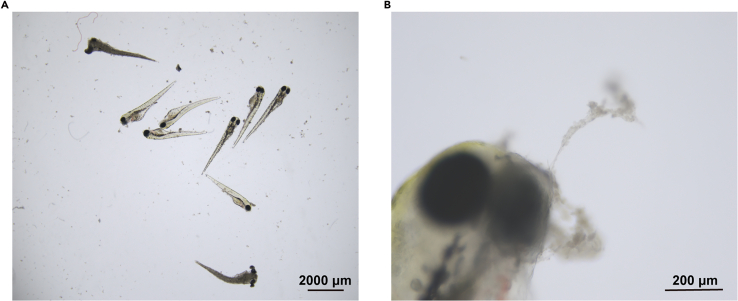
Physically impaired larvae (A) When 72 hpf zebrafish larvae were exposed to 150 μM AlCl_3_ solution for 48 hours without pH adjustment, extensive mortality is observed. Additionally, fungal growth developed on the surface of the larvae. Scale bar: 2000 μm. (B) Close-up view of fungal hyphae visibly growing on the larval surface. Scale bar: 200 μm.

**CRITICAL:** Precise pH control is critical for ensuring experimental validity. The pH of a 150 μM AlCl_3_ solution typically ranges from 4.5 to 4.6, a level that induces larval mortality (see Figure 2). Conversely, an excessively high pH reduces the concentration of Al^3+^.

**CRITICAL:** AlCl_3_·6H_2_O and NaOH are corrosive. Wear gloves and safety goggles.

**Table:** Behavior assay program setting

Time (Min)	Event
0–10	Light (light acclimatization)
10–20	Light
20–30	Dark
30–40	Light
40–50	Dark
50–60	Light
60–70	Dark

The behavioral experiment lasts 70 min, comprising a 10-minute light acclimatization period followed by three light/dark cycles, each consisting of 10 min of light and 10 min of darkness.

***Note:*** Exclude the movement data of zebrafish larvae recorded during the initial 10-min light adaptation period.

## Step-by-step method details

### Choose the larvae to be used for modeling

**Timing: 1–2 h (day 5)**

This protocol requires rigorous selection of developing zebrafish larvae designated for AD modeling based on standardized developmental staging criteria to ensure phenotypic uniformity.

1. Select zebrafish larvae of the same growth stage, and place them in a six-well plate, with 30 fish in each well.

***Note:*** We recommend starting this step at 9:00 A.M. and ensuring consistency across all experiments.

***Optional:*** If you want to evaluate the potential neuroprotective role of a candidate gene in Alzheimer's disease, you can generate transgenic zebrafish and then screen embryos for fluorescence signals such as EGFP at 2 dpf to confirm successful gene expression. If fluorescence is not observed in all embryos, select only those exhibiting fluorescence for further culture. Additionally, pick embryos with the same fluorescence intensity and exclude outliers to ensure experimental consistency.

### Treat larvae with AlCl_3_ solution

**Timing: 1–2 h per day for 3 consecutive days (days 5–7)**

This step involves establishing an AD model using the pre-prepared AlCl_3_ solution (150 μM AlCl_3_, pH 4.8–4.9).

***Note:*** We recommend starting step 2 and 3 immediately after step 1.

2. Aspirate the original 1× E3 medium from the six-well plate.
3. Add the pre-prepared AlCl_3_ solutions (150 μM AlCl_3_, pH 4.8–4.9), followed by a 72 h (3 days) treatment (Troubleshooting 1).

***Optional:*** For the validation of anti-Alzheimer’s disease drugs, an additional experimental group should be included in this step, in which the drug is mixed with AlCl_3_ solution (150 μM AlCl_3_, pH 4.8–4.9). Furthermore, if the drug requires dissolution in DMSO, a separate DMSO control group should also be established to account for any potential effects of the solvent.

***Note:*** If fungus is observed on the larvae during this step, terminate the experiment immediately.

***Note:*** During the modeling period, replace the pre-prepared AlCl_3_ solution with fresh solution every day (day 6 and 7).

**CRITICAL:** Complete removal of the E3 medium from the wells is not feasible, which may compromise the establishment of the model.

### Terminate the AlCl_3_ treatment

**Timing: 1 h (day 8)**

In this step, zebrafish larvae are transferred back to E3 medium.

4. Replace the E3 medium containing AlCl_3_ with E3 medium.
  a. Suck out the AlCl_3_ solution from the six-well plate.
  b. Wash each well twice with E3 medium to reduce the concentration of Al^3+^ in the solution.
  c. Add 3 mL of E3 medium into each well.

***Note:*** We recommend starting this step at 9:00 A.M.

### Perform behavioral assay

**Timing: 3–5 h (day 8 or day 9)**

This step involves terminating the treatment, followed by behavioral assessments to evaluate therapeutic outcomes.

***Note:*** Perform behavioral assays either on the day treatment ends (day 8) or on the following day (day 9).

***Note:*** We recommend starting this step at 10:00 A.M.

5. Add 1 mL of E3 medium to each well of a 48-well plate. Transfer one larva from the six-well plate to each well, ensuring only one larva per well.

***Note:*** Select zebrafish larvae that exhibit comparable activity levels to minimize the degree of dispersion in experimental outcomes.

**Figure 3:**
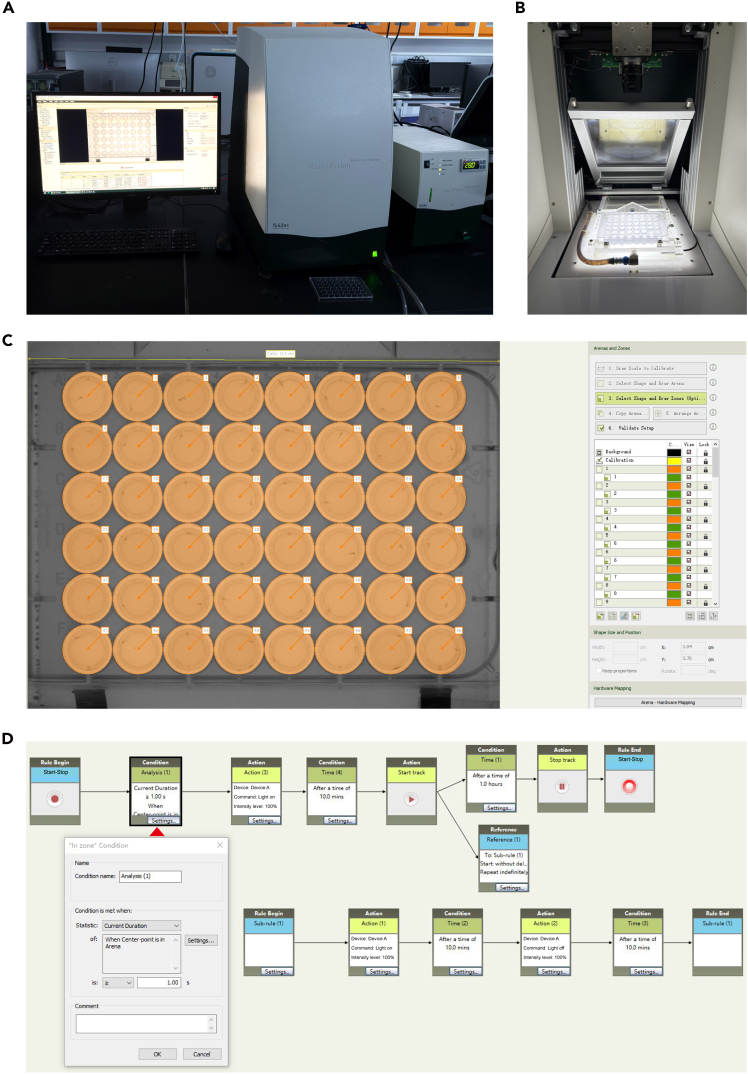
Behavioral instrument and key program settings (A) Overview of the behavioral apparatus. (B) Imaging module of the behavioral apparatus, showing the 48-well plate. (C) Configuration of the detection regions and distance reference in the software. (D) Setup of the light/dark cycle program for behavioral analysis.
7. Remove the lid, and place the 48-well plate containing the larval into the behavioral instrument (Figure 3B).
8. Configure a behavioral testing procedure (Figures 3C and 3D).
  a. Define the detection region as a circular area (Figure 3C).
  b. Set the length of the 48-well plate as the reference, which is 12.5 cm (Figure 3C).
  c. Set up an automated light/dark cycle program (Figure 3D).
  d. Adjust the recognition sensitivity until each moving larva can be accurately identified (Troubleshooting 2).

***Note:*** Ensure that the circular area completely covers each well of the 48-well plate.

***Note:*** During the dark phase of the light/dark cycle, the DanioVision system combines infrared (IR) illumination with an infrared-sensitive camera to enable tracking in darkness. The system uses infrared wavelengths that are invisible to zebrafish, thereby avoiding measurement bias, ensuring accurate image acquisition, and eliminating the need for additional setup.

6. Turn on the behavioral testing equipment and computer, set the temperature to 28 °C (Figure 3A), and allow it to reach the set point.

9. Initiate the behavioral assay using the pre-configured program settings.
10. After the behavioral test is completed, export the required data and figures (Troubleshooting 3).
  a. Export the data containing per-minute and total measurements, ensuring each dataset includes parameters such as distance and velocity.
  b. Export the digital track maps (the first 10 min).

***Note:*** You can export the track map for 1 h; however, it may not exhibit any noticeable differences.

**CRITICAL:** As zebrafish larvae behavior is regulated by the circadian clock, conducting behavioral assessments between 10:00 and 15:00 is optimal.^14^

## Expected outcomes

**Figure 4:**
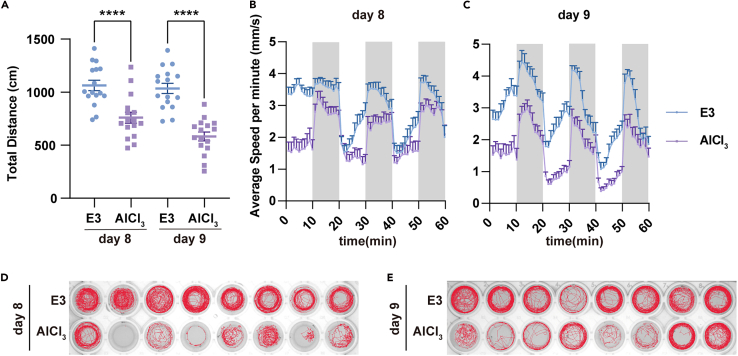
Expected results in behavioral analysis AlCl_3_ treatment is administered from 72 hpf to 144 hpf (A) Total distance traveled by zebrafish larvae in the E3 (control) and AlCl_3_ modeling groups within 60 min at 144 hpf and 144+24 hpf. Data are shown as mean ± S.E.M. (n = 16). Statistical analysis was performed using two-way ANOVA followed by Tukey’s post hoc test, with significance indicated as ∗∗∗∗*p* < 0.0001 between the E3 and AlCl_3_ groups. (B) and (C) Average movement speed per minute of zebrafish larvae in E3 and AlCl_3_ modeling groups during a 60-minute observation period at 144 hpf and 144+24 hpf developmental stages. (D) and (E) The digital track map of zebrafish larvae during the first 10 min after the start of the experiment at 144 hpf (D) and 144+24 hpf (E).

Behavioral analysis in zebrafish larvae showed that, compared with the non-model group, larvae in the Alzheimer’s disease (AD) model group exhibited significantly reduced total swimming distance (Figure 4A) and average swimming speed per minute (Figures 4B and 4C). Trajectory plots from the first 10 min (recorded after a 10-minute light adaptation period) also clearly demonstrated that, on day 8 (Figure 4D) and day 9 (Figure 4E), larvae in the AD model group traveled shorter distances than those in the E3 group. These results indicate that, compared with the control group (E3), the AlCl_3_-treated AD model group shows impaired locomotor activity and reduced responsiveness to light/dark cycles. In summary, our protocol effectively recapitulates AD-related phenotypes with minimal toxicity to the larvae, offering a robust platform for high-throughput AD drug screening. See also Table S1.

## Quantification and statistical analysis

All experimental data are expressed as the mean ± standard error of the mean (SEM). Statistical analyses were conducted using GraphPad Prism software, with intergroup comparisons assessed by two-way analysis of variance (ANOVA) followed by Tukey’s post hoc test. Statistical significance was defined as ∗*p* < 0.05, ∗∗*p* < 0.01, ∗∗∗*p* < 0.001, and ∗∗∗∗*p* < 0.0001.

## Limitations

Experimental consistency across trials may be limited by biological variability in zebrafish spawning timing and embryo quality. Maintaining stable pH levels during AlCl_3_ exposure poses significant technical challenges, particularly when compounded by potential inaccuracies in pH meter measurements. Instability in pH may induce unintended physiological stress or lead to model failure, thereby undermining the experimental validity. The reliability of behavioral data may be compromised by potential inaccuracies arising from equipment calibration errors or the lack of specialized instruments. Experiments involving live animals can be unpredictable, and zebrafish larvae may exhibit variable responsiveness to light/dark cycle transitions or exposure to chemical agents due to unidentified factors. Although this protocol enables large-scale AD modeling in a short period, the results can only be considered preliminary due to the evolutionary distance between zebrafish and humans.

## Troubleshooting

### Problem 1

Completely removing the 1× E3 medium from the 6-well plate is a challenging task. Incomplete removal may result in dilution of the AlCl_3_ solution, thereby compromising the establishment of the model. (related to Step 3).

### Potential solution

Wash with the AlCl_3_ solution (150 μM AlCl_3_, pH 4.8–4.9) at least two times.

### Problem 2

The movement distance or activity level of zebrafish larvae is relatively low during behavioral assays (related to Step 8).

### Potential solution

If zebrafish larvae exhibit visible swimming activity but recorded movement distances are significantly low, proceed as follows: save the current video recording, adjust the detection sensitivity settings, and re-run the behavioral test using the saved video.

### Problem 3

Inaccurate pH meter readings can cause the pH value to deviate from the target range, thereby affecting the accuracy of the AD model, meaning the behavioral results do not show differences between the modeled and non-modeled groups. (related to Step 10).

### Potential solution

If behavioral assays indicate unsuccessful AD modeling, lower the pH of the solution. If zebrafish larvae exhibit mortality or external fungal growth, remove the affected larvae and repeat the experiment using a fresh AlCl_3_ solution with a moderately increased pH.

## Resource availability

### Lead contact

For further information or requests related to resources and reagents, please contact the lead investigator, Ruiqin Hu (rqhu@shou.edu.cn), who will be responsible for fulfilling these inquiries.

### Technical contact

Technical questions on executing this protocol should be directed to and will be answered by the technical contacts, Zequan Lin (17701789207@163.com), Lufang Zou (1853784820@qq.com), and Wei Li (e1998liwei@163.com).

### Materials availability

This study did not generate any new unique reagents.

### Data and code availability

The data generated in this study are included in the manuscript. For additional information or inquiries, please contact the corresponding author.

## Acknowledgments

The work is supported by the SciTech Funding by CSPFTZ Lingang Special Area Marine Biomedical Innovation Platform (no.YF251101 to L.C.).

## Author contributions

R.H. and L.C. conceived and supervised the study. Z.L., L.Z., and W.L. optimized the protocol, analyzed the data, and wrote the initial draft. L.P. and J.Z. helped with the revision and figure illustration. R.H. and L.C. edited and finalized the manuscript.

## Declaration of interests

The authors declare no competing interests.

## Supplemental information

Table S1. Raw data of expected results in behavioral analysis, related to Expected Outcomes

## References

[1] Sande R., Godad A., Doshi G. (2024). Zebrafish Experimental Animal Models for AD: A Comprehensive Review. Curr. Rev. Clin. Exp. Pharmacol..

[2] Lepesant J.-A. (2015). The promises of neurodegenerative disease modeling. C. R. Biol..

[3] Chen X., Barclay J.W., Burgoyne R.D., Morgan A. (2015). Using C. elegans to discover therapeutic compounds for ageing-associated neurodegenerative diseases. Chem. Cent. J..

[4] Tello J.A., Williams H.E., Eppler R.M., Steinhilb M.L., Khanna M. (2022). Animal Models of Neurodegenerative Disease: Recent Advances in Fly Highlight Innovative Approaches to Drug Discovery. Front. Mol. Neurosci..

[5] Chen H., Li H., Yin X., Liu Y., Zhang T., Wu H., Kang G., Yu Y., Bai M., Bao L. (2023). The therapeutic effect of Zhenbao pills on behavioral changes in zebrafish caused by aluminum chloride. Biomed. Pharmacother..

[6] Pan H., Zhang J., Wang Y., Cui K., Cao Y., Wang L., Wu Y. (2019). Linarin improves the dyskinesia recovery in Alzheimer's disease zebrafish by inhibiting the acetylcholinesterase activity. Life Sci..

[7] Uvarajan D., Ravikumar M., Durairaj B. (2026). Arbutin attenuates aluminium chloride–induced neurotoxicity and cognitive deficits in a zebrafish model of Alzheimer’s disease. Naunyn-Schmiedebergs Arch Pharmacol.

[8] Vaja R., Vohra M., Ramachandran A.V., Baxi D. (2025). Development of a Novel Aluminium Chloride-Induced Zebrafish Model of Alzheimer's Disease: Involvement of Oxidative Stress, Cholinergic Dysfunction, and Gut Pathophysiology. Neurotox. Res..

[9] Wu Y., Gao Y., Tie F., Wang R., Hu N., Dong Q., Fu C., Wang H. (2025). The Neuroprotective Effects of Cyanidin Derivatives on AlCl(3)-Induced Zebrafish Model of Alzheimer's Disease. Molecules.

[10] Huang W., Li C., Shen Z., Zhu X., Xia B., Li C. (2016). Development of a Zebrafish Model for Rapid Drug Screening against Alzheimer’s Disease. bioRxiv.

[11] Uvarajan D., Durairaj B. (2023). Arbutin aids in the recovery of dyskinesia in Alzheimer's zebrafish by decreasing the function of acetylcholinesterase. Res. J. Biotechnol..

[12] Sayer M.D.J., Reader J.P., Dalziel T.R.K. (1993). Freshwater acidification: effects on the early life stages of fish. Rev. Fish Biol. Fish..

[13] Brown P.L., Sylva R.N., Batley G.E., Ellis J. (1985). The hydrolysis of metal ions. Part 8. Aluminium(III). J. Chem. Soc. Dalton Trans..

[14] Tagkalidou N., Multisanti C.R., Bleda M.J., Bedrossiantz J., Prats E., Faggio C., Barata C., Raldúa D. (2024). Analyzing the Effects of Age, Time of Day, and Experiment on the Basal Locomotor Activity and Light-Off Visual Motor Response Assays in Zebrafish Larvae. Toxics.

